# Proteolysis of Micellar β-Casein by Trypsin: Secondary Structure Characterization and Kinetic Modeling at Different Enzyme Concentrations

**DOI:** 10.3390/ijms24043874

**Published:** 2023-02-15

**Authors:** Mikhail M. Vorob’ev, Burçin Dersu Açıkgöz, Günnur Güler, Andrey V. Golovanov, Olga V. Sinitsyna

**Affiliations:** 1 A.N. Nesmeyanov Institute of Organoelement Compounds, RAS, 28 ul. Vavilova, 119991 Moscow, Russia; 2 Division of Bioengineering, Graduate School, Izmir University of Economics, Izmir 35330, Turkey; 3 Biophysics Laboratory, Department of Physics, Izmir Institute of Technology, Urla, Izmir 35430, Turkey; 4 Biomedical Bioengineering, Izmir University of Economics, Sakarya Cad., Izmir 35330, Turkey

**Keywords:** proteolysis kinetics, Fourier-transform infrared spectroscopy, atomic force microscopy, beta-casein, trypsin

## Abstract

Tryptic proteolysis of protein micelles was studied using β-casein (β-CN) as an example. Hydrolysis of specific peptide bonds in β-CN leads to the degradation and rearrangement of the original micelles and the formation of new nanoparticles from their fragments. Samples of these nanoparticles dried on a mica surface were characterized by atomic force microscopy (AFM) when the proteolytic reaction had been stopped by tryptic inhibitor or by heating. The changes in the content of β-sheets, α-helices, and hydrolysis products during proteolysis were estimated by using Fourier-transform infrared (FTIR) spectroscopy. In the current study, a simple kinetic model with three successive stages is proposed to predict the rearrangement of nanoparticles and the formation of proteolysis products, as well as changes in the secondary structure during proteolysis at various enzyme concentrations. The model determines for which steps the rate constants are proportional to the enzyme concentration, and in which intermediate nano-components the protein secondary structure is retained and in which it is reduced. The model predictions were in agreement with the FTIR results for tryptic hydrolysis of β-CN at different concentrations of the enzyme.

## 1. Introduction

The classical models of proteolysis, such as the Linderström-Lang model [1] or the exponential model [2,3], consider the hydrolysis of peptide bonds without indicating their localization either in soluble single molecules or in protein associates. This leads to an inaccurate description of proteolysis, since the rate of hydrolysis of peptide bonds in soluble polypeptides and the same peptide bonds hidden in aggregates, is different. This problem was partially solved in the two-step proteolysis model [4], in which masked peptide bonds could be hydrolyzed only after their demasking. The masked peptide bonds are located inside protein globules or in the aggregates where their enzymatic attack is hindered. Assuming that the masked bonds are transformed to the demasked ones during proteolysis, the description of proteolysis can be brought closer to reality [4,5].

An important example of the proteolysis of a protein substrate in associated form is the proteolysis of protein micelles. In the current publication, we present the kinetic analysis of the proteolysis of β-casein (β-CN) micelles by trypsin, in addition to our recent study of the tryptic hydrolysis of β-CN at low concentrations using the two-step proteolysis model [6,7]. Herein, we explicitly took into account the process of the degradation and rearrangement of β-CN micelles at various enzyme concentrations and constant substrate concentration.

Proteolysis of milk-casein micelles, consisting of α_S1_-, β-, and κ-caseins, was intensively studied in connection with the analysis of the factors that determine the stability of these micelles and, consequently, the shelf life of milk [8,9,10]. Much attention in these studies was given to the experimental determination of the size distribution, zeta potential, and hydration of the micelles depending on various destabilizing factors. In addition to being used as food, the various nanoforms of milk casein are promising for targeted drug delivery and tissue engineering [11]. β-CN micelles are of interest as a convenient object for physicochemical studies [12,13] and as a potential carrier of hydrophobic drugs [14,15]. The proteolysis of milk-casein micelles by chymosin is well studied. In this process, the κ-casein located on the surface of the micelles is first hydrolyzed, which causes destabilization of the micelles, their coagulation, and the formation of a curd [8]. Proteolysis of β-casein micelles has been studied less, mainly to understand the hydrolysis kinetics of specific peptide bonds cleaved by native [16,17] or engineered trypsin [18,19].

The fractal model of the aggregation of protein colloidal particles [20], in principle, can be used to describe the aggregation of destabilized milk-casein micelles. For this, the hydrolysis of one key peptide bond of κ-casein, which destabilizes the milk-casein micelle, should be considered, while the hydrolysis of other peptide bonds can be neglected. For the proteolysis of β-CN micelles by trypsin, this assumption is incorrect, since the hydrolysis of a set of peptide bonds and the aggregation of peptide fragments are interrelated and cannot be separated from each other. In general, physicochemical models are difficult to apply to the proteolysis of amphiphilic proteins due to the extreme complexity of this phenomenon.

β-casein (β-CN), the amphiphilic protein (oligopeptide) with 209 amino acid residues, forms associates and soap-like micelles [21,22]. During the hydrolysis of peptide bonds in β-CN, both the degradation of the initial β-CN micelles and the formation of new micelles occur [23]. A complete description of the rearrangement of micelles is hampered by the need to analyze a large number of particles of different sizes, and knowing only the average values of their diameters is insufficient for such a description [23]. An important issue that remains almost unexplored is a change of the secondary structure of the micellar proteins in the course of proteolysis.

Formerly, we studied the kinetics of β-CN hydrolysis (proteolysis) by bovine trypsin (EC 3.4.21.4) in comparison with hydrolysis of globular β-lactoglobulin [5]. The substrate concentration of 0.25 g/L [5,24,25] was below the critical micelle concentration (CMC) of the β-CN solution (0.5–1 g/L), and before the action of trypsin the solution was not micellar. At this condition, the proteolysis of β-CN by trypsin caused the formation of nanoparticles consisting of the products of enzymatic hydrolysis [24,25]. For the first time, this process was demonstrated by the method of static light-scattering dissymmetry (Debye method), which ensures continuous recording during the proteolysis reaction of the concentration of nanoparticles and their sizes in the range of 10–200 nm [24,25].

At the β-CN concentration of 3 g/L greater than the CMC of a β-CN solution, it was shown that the obtained micelles (peptide nanoparticles [23]) are different from the original casein micelles, which are soap-like micelles [26]. Analysis of these nanoparticles dried on the surfaces of mica and graphite by using atomic force microscopy (AFM) made it possible to estimate their size, density, and structural heterogeneity [23,27]. The obtained nanoparticles were found to be approximately two times denser than initial casein micelles. To elucidate the details of the mechanism of the formation and further degradation of β-CN particles, it is necessary to fully characterize the set of particles, i.e., determine the particle-size distribution functions [23].

The percentage of the hydrolyzed peptide bonds (degree of hydrolysis) is used by proteolysis researchers to control and optimize the proteolysis process [4,28]. An alternative to the degree of hydrolysis is spectroscopic methods that allow online continuous recording. The possibility of monitoring the course of proteolysis has been shown by using fluorescence [5,24], infrared (FTIR) [29,30], and ultrasonic [31,32] spectroscopic methods.

FTIR spectroscopy, a well-established technique, has long been widely used for biophysical and biochemical studies, including analysis of the secondary structure and conformational changes in biological macromolecules [33,34,35,36,37]. Recently, the alterations in the secondary structures of globular and non-globular proteins and the time course of their changes during proteolytic reaction have been compared in our studies at higher concentrations of the enzyme than in the present study [29,30]. We found that regular secondary protein structures (β-sheet and α-helix) in the amide I band (1700–1600 cm^−1^) were reduced, while unordered structures as well as digestion products increased (the intensity ∼1594 cm^−1^ and ∼1406 cm^−1^ was due to the antisymmetric and symmetric stretching modes of free carboxylates, respectively), as the proteolysis proceeded.

The hydrolysis of β-CN with accumulation and decay of intermediate nanoparticles was previously described by a simple kinetic scheme with two stages and one intermediate component, which made it possible to explain the sharp increase in the number of nanoparticles recorded using static light scattering at 45° [24]. A detailed scheme of the rearrangement of micelles should include more stages, but on the other hand, an overly complex scheme is difficult to substantiate experimentally. As a reasonable compromise, we examine herein a three-step successive scheme for which the time dependences of the concentrations of components can be calculated relatively simply.

The purpose of the current study was to investigate the proteolysis of β-CN by trypsin and to determine the nanoparticle rearrangement and the changes in protein secondary structure by using AFM, FTIR, and the methods of chemical kinetics.

## 2. Results

### 2.1. Characterization of Nanoparticles by Atomic Force Microscopy

**Figure 1:**
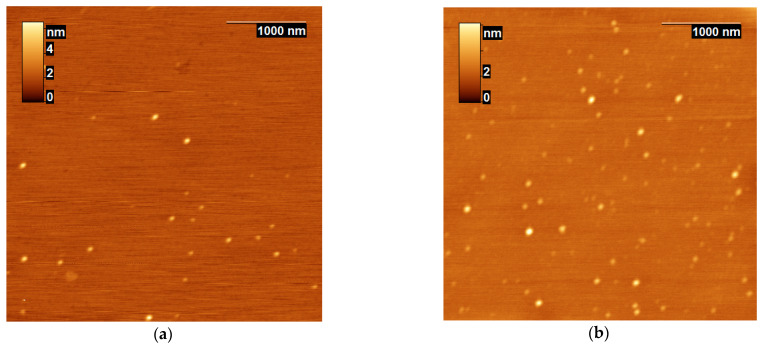
Topographic images of β-CN nanoparticles digested with trypsin for 90 min, followed by heat inactivation of trypsin activity (**a**, sample 1) or inaction of trypsin activity with soybean trypsin inhibitor (**b**, sample 2).

The analyzed nano-components of the reaction mixture (nanoparticles) were collected from the hydrolyzed β-CN by trypsin in aqueous phosphate buffer. The serine protease provides cleavage of Arg-X and Lys-X peptide bonds, while the number of hydrolyzed bonds (degree of hydrolysis) can be varied by changing the hydrolysis time, the reagent concentrations, and the physicochemical conditions of proteolysis. The presented example of the nanoparticles (Figure 1) was obtained for the hydrolysis duration of 90 min at the β-CN concentration of 3 g/L and trypsin concentration of 1 mg/L. Termination of the hydrolysis of peptide bonds by enzyme inhibition with a soybean trypsin inhibitor or by heat inactivation resulted in time-stable particles (Figure 1a,b).

**Figure 2:**
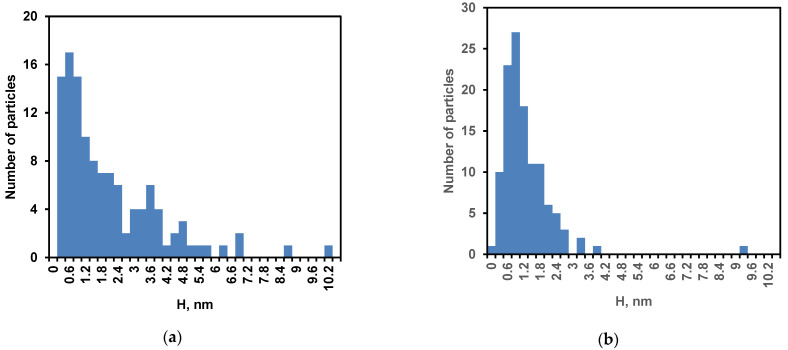
Height distribution of nanoparticles: (**a**) sample 1 obtained with heat inactivation of trypsin and (**b**) sample 2 obtained by inhibiting the enzyme with soybean trypsin inhibitor.

**Figure 3:**
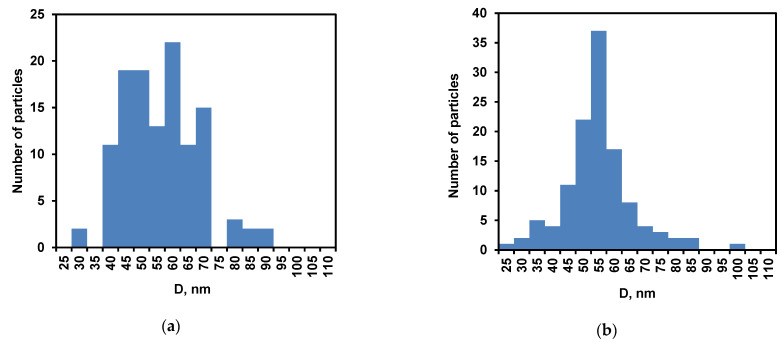
Diameter distribution of nanoparticles: (**a**) sample 1 obtained with heat inactivation of trypsin and (**b**) sample 2 obtained by inhibiting the enzyme with soybean trypsin inhibitor.

The samples after heat treatment (sample 1) or addition of the soy inhibitor (sample 2) were placed on the mica surface. After drying, the particles were analyzed on the mica surface, and for each of the particles its height (H) and diameter (D) were determined (Figure 2). The particle height distribution for sample 1 was broad and bimodal with peaks at 0.8 and 3.8 nm (Figure 2a). Sample 2 had a unimodal particle height distribution with a maximum at 1 nm (Figure 2b). The mean particle diameter was 58 ± 12 nm and 57 ± 12 nm for samples 1 and 2, respectively (Figure 3).

In addition to the well-known particle broadening due to the AFM probe, it can be assumed that the shape of the particles was flattened as a result of their interaction with the mica surface during adsorption and subsequent drying [27]. The mean particle volumes were estimated as 3500 nm^3^ and 2200 nm^3^ in samples 1 and 2, which corresponds to the volumes of spherical particles with the radii of 9.4 nm and 8.1 nm, respectively. The heating led to an increase in the size of nanoparticles, probably due to additional aggregation [38]. This can also explain a decrease in the number of nanoparticles in Figure 1a compared to Figure 1b.

Thus, regardless of the method of sample preparation, the AFM method confirms the formation of the nanoparticles of different sizes as a result of proteolysis of *β*-CN by trypsin. This result is consistent with our data comparing the original micelles of *β*-CN with nanoparticles obtained with stopping the reaction by soybean inhibitor [23,27]. However, any method of the stopping the reaction or sampling manipulation introduces changes in the particle distribution, as shown by comparing samples 1 and 2, for example. Without stopping the reaction and sampling, it is difficult to use the AFM method for determining the complete distribution of nanoparticles, since this distribution changes during proteolysis. This reduces the possibility of using this method to build a quantitative model of proteolysis. Therefore, we carried out further studies without sampling, studying the rearrangement of particles directly in the temperature-controlled cuvette of the FTIR spectrometer.

### 2.2. Characterization of Proteolysis of β-CN by Trypsin with Infrared Spectroscopy

In the current study, the hydrolysis of β-CN by trypsin at relatively low concentrations of the enzyme compared to our previous work [29,30] was characterized by FTIR spectroscopy. Herein, the β-CN and trypsin solutions were prepared in deuterated buffer (20 mM potassium phosphate buffer at pD 7.9) after overnight deuteration at +4 °C. The β-CN concentration in the proteolysis reaction at temperature of 37 °C was 25 g/L, while *S*_0_/*E*_0_ ratios were 500, 4000, and 10,000 (*w*/*w*).

**Figure 4:**
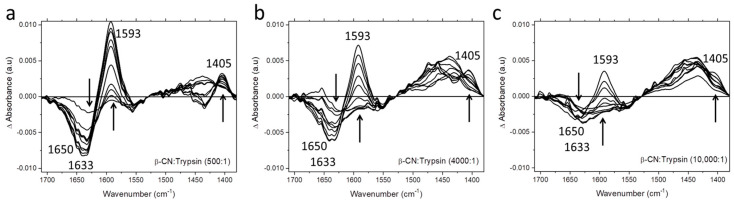
The FTIR difference spectra for β-CN digested by trypsin recorded at 37 °C for the *S*_0_/*E*_0_ ratios of (**a**) 500, (**b**) 4000, and (**c**) 10,000 (*w*/*w*). The difference spectra were calculated by subtraction of the first spectrum recorded at *t* = 1 min from each of the absorbance spectra recorded during enzymatic reaction. The arrows represent the amide changes of β-CN during digestion as a function of time from 1 min to 90 min.

Based on the FTIR-difference spectra for all β-CN to trypsin ratios probed herein (Figure 4), protein secondary structures (such as β-sheets at 1633 cm^−1^ and α-helices at 1650 cm^−1^) undergo significant amide changes while the intensities at 1593 cm^−1^ and 1405 cm^−1^ increase (due to antisymmetric and symmetric stretching of free carboxylates, respectively), as the enzymatic reaction proceeds for 90 min. The spectral changes were observed to a lesser extent when the trypsin concentration was too low at *S*_0_/*E*_0_ = 10,000.

**Figure 5:**
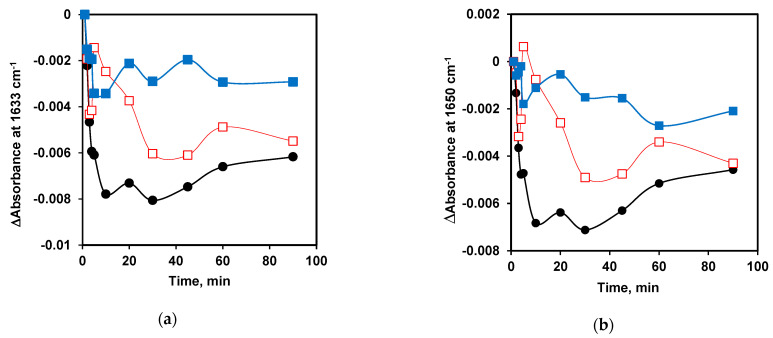

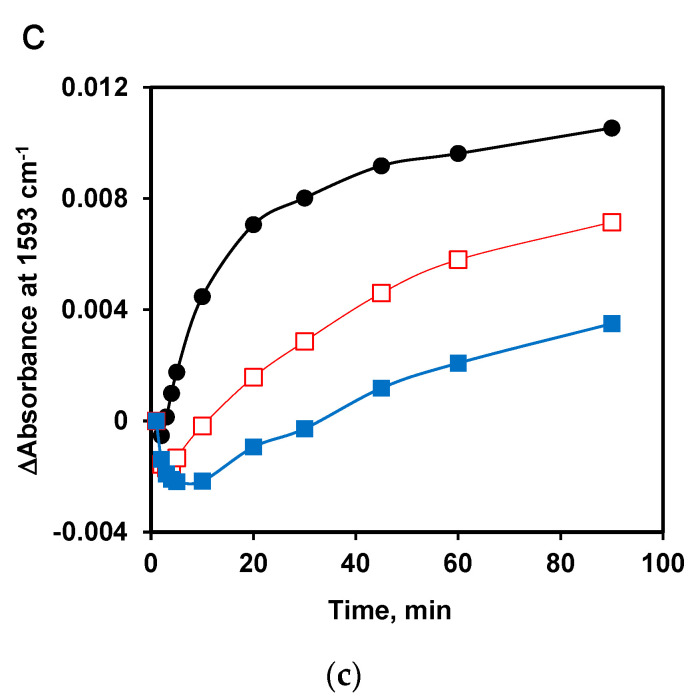
The intensity changes obtained from the FTIR-difference spectra (from Figure 4) with the *S*_0_/*E*_0_ ratios of 500 (●, black), 4000 (□, red), and 10,000 (■, blue) as a function of time during proteolysis reaction. The content of (**a**) β-sheets absorbing at 1633 cm^−1^, (**b**) α-helix absorbing at 1650 cm^−1^, and (**c**) antisymmetric stretching of free carboxylates (proteolysis products) absorbing at 1593 cm^−1^.

Figure 5 shows the intensity changes (Δ absorbance or absorbance difference) at 1633 cm^−1^ (due to β-sheet) and 1650 cm^−1^ (due to α-helix), as well as at 1593 cm^−1^ (antisymmetric stretching of free carboxylates as proteolysis products). These time-dependences were obtained from the FTIR-difference spectra (shown in Figure 4) for proteolytic reactions carried out at *S*_0_/*E*_0_ ratios of 500, 4000, and 10,000. The main trend was that the relative content of β-sheet and α-helical structures decreased while the amount of free carboxylate groups as proteolysis products increased upon enzymatic reaction. A high amount of enzyme at *S*_0_/*E*_0_ = 500 generates more changes, causing a larger reduction in the content of both β-sheets and α-helices of β-CN (Figure 5a,b) and a larger increase in the content of free carboxylate groups released due to the hydrolysis of specific peptide bonds by trypsin (Figure 5c).

However, at a lower enzyme concentration (*S*_0_/*E*_0_ = 10,000) the changes in the content of β-sheets and α-helical structures during proteolysis were smaller and the reactions slower (Figure 5). In the early phase of hydrolysis, for instance, the β-sheet content decreased rapidly (within first 5 min), but subsequently its relative content increased and then decreased again, forming a local maximum. The proteolysis products (at 1593 cm^−1^) began to increase only after 10 min from the start of the process. This clearly indicates that the peptide nanoparticles of β-CN were rearranged some time after the onset of proteolysis and the release of hydrolysis products began after a lag phase. For *S*_0_/*E*_0_ = 4000, similar regularities in the content of β-sheets and α-helices during proteolysis were also observed but reduction in the content of secondary structures was larger and faster (first 4 min) in comparison to the case at *S*_0_/*E*_0_ = 10,000.

### 2.3. Kinetic Model of the Rearrangement of Particles during β-CN Proteolysis by Trypsin

We introduced three rate constants *k_I_*, *k_II_*, and *k_III_* for the following three-step successive scheme of the proteolysis of micellar casein: original micelles $\overset{k_{I}}{\to }$ micelles with hydrolyzed bonds $\overset{k_{II}}{\to }$ new nanoparticles $\overset{k_{III}}{\to }$ low-weight peptide fragments or aggregates of new nanoparticles. If all three rate constants are strictly proportional to the enzyme concentration, the kinetics is simple. In this case, the time of particle formation *t*_max_ should be inversely proportional to the enzyme concentration *E*_0_ (*t*_max_~1/*E*_0_), i.e., the dependence *t*_max_ on *E*_0_ is a simple hyperbola. However, our previous studies have shown that this dependence is not a hyperbola [24], and hence a more complex model needs to be proposed.

**Figure 6:**
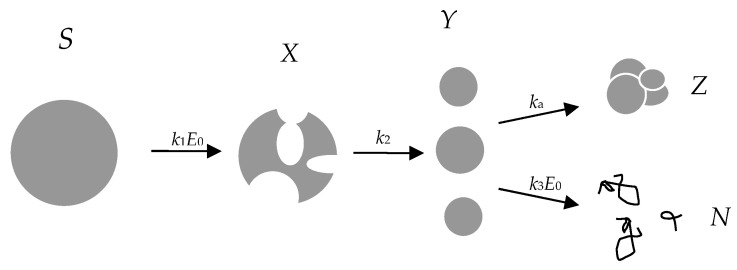
Successive scheme of β-CN proteolysis by trypsin describing rearrangement of peptide nanoparticles.

In the current study, a three-step successive scheme (Equation (1) and Figure 6) is considered in which the constants of the first and third stages are proportional to *E*_0_, and the rate constant of the second stage does not depend on *E*_0_:

(1) $$\begin{array}{l} S\overset{k_{1}E_{0}}{\to }X\overset{k_{2}}{\to }Y\overset{k_{3}E_{0}}{\to }N\\ \begin{array}{ccc} & k_{a}\begin{array}{cc} ↓ & \end{array} & \end{array}\\ \begin{array}{cc} \begin{array}{cc} & \begin{array}{cc} & Z \end{array} \end{array} & \end{array} \end{array},$$

where *S* stands for the initial micelle with intact β-CN molecules, *X* stands for the micelle with hydrolyzed peptide bonds, *Y* stands for the new nanoparticles obtained on the basis of the hydrolyzed micelle, *Z* stands for the large nanoparticle obtained by aggregation of *Y* nanoparticles, and *N* stands for the peptide products of the hydrolysis of β-CN.

It is assumed that micelles *X* with hydrolyzed peptide bonds include hydrophobic centers that serve as nuclei for the assembly of new particles *Y*. In addition to the degradation of nanoparticles to peptides *N* not detected by AFM or light scattering, the third stage also contains an additional aggregation process, leading to the formation of large nanoparticles *Z* with diameter higher than 100 nm. Such large nanoparticles, which are formed with the rate constant *k*_a_, as shown in Figure 6, were found in a relatively small amount compared to other particles [23]. For simplicity, in the model considered here, *k*_a_ and *Z* are assumed to be equal to 0. This simplification is reasonable for short proteolysis times when the aggregates do not have time to form.

The differential equations corresponding to the three-step successive scheme of proteolysis can be solved analytically assuming that the concentration of the active enzyme is constant. In particular, the dependence of the concentration of the nanoparticles *Y* on the time of proteolysis is expressed by the following equation (*S*_0_ = 1):

(2) $$Y(t)=k_{1}E_{0}k_{2}\left[\frac{e^{-k_{1}E_{0}^{}t}}{\left(k_{2}-k_{1}E_{0}\right)\left(k_{3}E_{0}-k_{1}E_{0}\right)}+\frac{e^{-k_{2}t}}{\left(k_{2}-k_{3}E_{0}\right)\left(k_{2}-k_{1}E_{0}\right)}+\frac{e^{-k_{3}E_{0}t}}{\left(k_{3}E_{0}-k_{1}E_{0}\right)\left(k_{3}E_{0}-k_{2}\right)}\right]$$

This function first increases and then decreases during proteolysis, so that the time *t*_max_ at which the maximum of this function occurs can be calculated at the point when d*Y*/d*t* = 0 by solving the following transcendental equation:

(3) $$k_{1}E_{0}\left(k_{3}E_{0}-k_{2}\right)e^{-k_{1}E_{0}t_{\max}}-k_{2}\left(k_{3}E_{0}-k_{1}E_{0}\right)e^{-k_{2}t_{\max}}-k_{3}E_{0}\left(k_{1}E_{0}-k_{2}\right)e^{-k_{3}E_{0}t_{\max}}=0$$

Equation (3) can also be used to calculate the constant *k*_2_, if the values of *t*_max_, *E*_0_, *k*_1_, and *k*_3_ are known.

**Figure 7:**
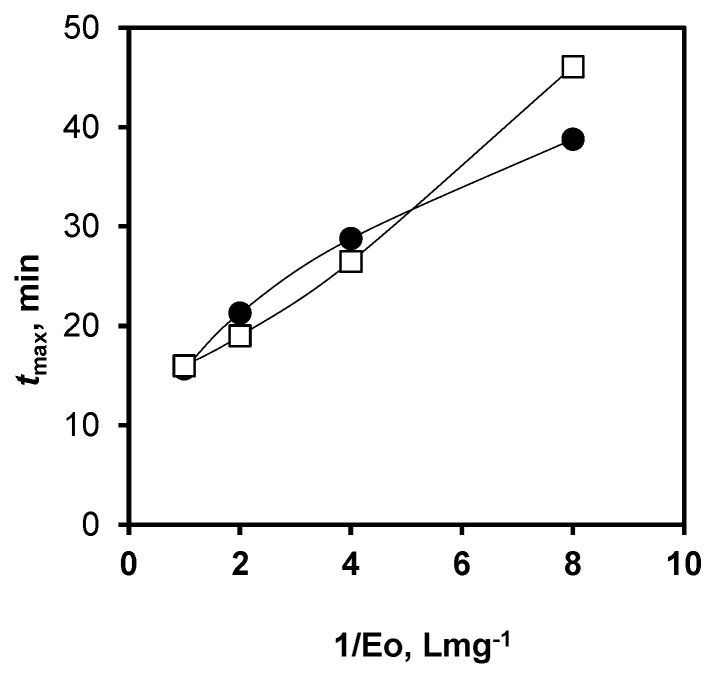
Experimental (□) and calculated (●) values of *t*_max_ depending on the reciprocal values of enzyme concentration.

The value of the rate constant *k*_1_ was taken from the determination of the hydrolysis rate at the beginning of hydrolysis [24], so *k*_1_*E*_0_ = 0.0066 s^−1^ for *E*_0_ = 0.25 mg/L. The rate constant *k*_3_ was estimated formerly from the fluorescence and light-scattering data at long hydrolysis times [24]. From these data, *k*_3_*E*_0_ was taken to be 0.0002 s^−1^ for *E*_0_ = 0.25 mg/L. The experimental values of *t*_max_ for *E*_0_ = 1, 0.5, 0.25, and 0.125 mg/L were used [24]. The fitted value of the rate constant *k*_2_ = 0.0015 s^−1^ was used to calculate the theoretical dependence of *t*_max_ on 1/*E*_0_, which follows from the transcendental Equation (3). The similarity between the experimental and calculated values of *t*_max_ is shown in Figure 7.

**Figure 8:**
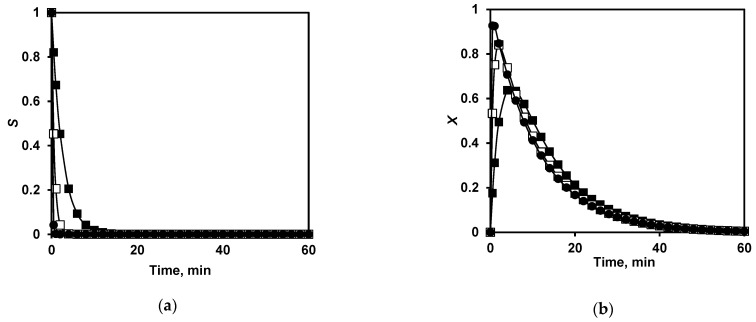

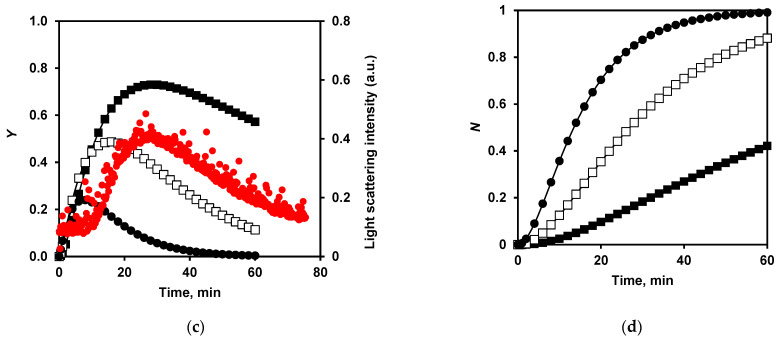
Calculated time dependences for functions (**a**) *S*(*t*), (**b**) *X*(*t*), (**c**) *Y*(*t*), and (**d**) *N*(*t*) for proteolysis processes at different enzyme concentrations: *E*_0_ = 0.25 mg/L, *S*_0_/*E*_0_ = 1000 (■); *E*_0_ = 1 mg/L, *S*_0_/*E*_0_ = 250 (□); and *E*_0_ = 4 mg/L, *S*_0_/*E*_0_ = 63 (●). (**c**) Experimental points for light-scattering data (●, red) at *E*_0_ = 0.25 mg/L and *S*_0_/*E*_0_ = 1000.

The obtained values of the rate constants *k*_1_, *k*_2_, and *k*_3_ were used to calculate the changes of the concentrations *S*(*t*), *X*(*t*), *Y*(*t*), and *N*(*t*) during proteolysis (Figure 8). The dependences calculated with Equation (2) for *Y(t*) at different *S*_0_/*E*_0_ ratios (Figure 8c) were close to those obtained by static light scattering for the proteolysis of β-CN at different trypsin concentrations (Figure 1 in [24]). One of these experimental curves for *E*_0_ = 0.25 mg/L is shown in Figure 8c. *X*(*t*) and *Y*(*t*) describe changes in the concentrations of intermediate products, which are first accumulated and then decayed. *S*(*t*) decreases from the beginning of the reaction, while *N*(*t*) grows with some delay in time (lag phase). The larger the *S*_0_/*E*_0_ parameter, the larger are *t*_max_ and the lag value (Figure 8).

**Figure 9:**
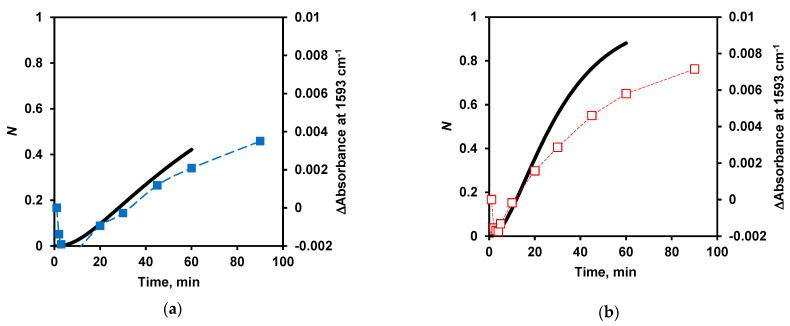

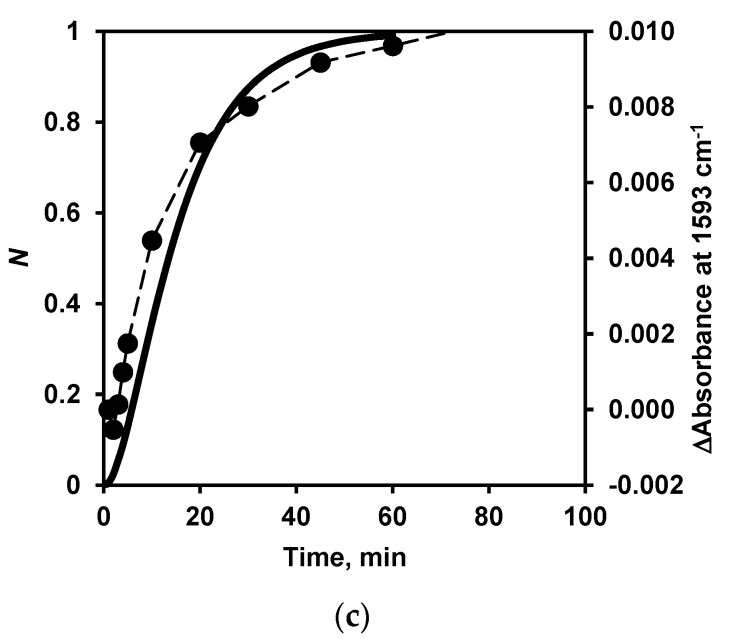
Yield of proteolysis products *N*(*t*) according to the model (solid line) at *S*_0_/*E*_0_ ratios of 1000 (**a**), 250 (**b**), and 63 (**c**) and FTIR data (dotted line) at *S*_0_/*E*_0_ ratios of 10,000 (**a**, ■, blue), 4000 (**b**, □, red), and 500 (**c**, ●, black).

The dependences *N(t*) were close to the dependences describing the growth of free carboxylate groups measured at 1593 cm^−1^ (Figure 9). At low trypsin concentration, hydrolysis products were formed with a lag phase, which was predicted in the proteolysis reactions theoretically [39] and observed experimentally by FTIR spectroscopy in the current study (Figure 9).

**Figure 10:**
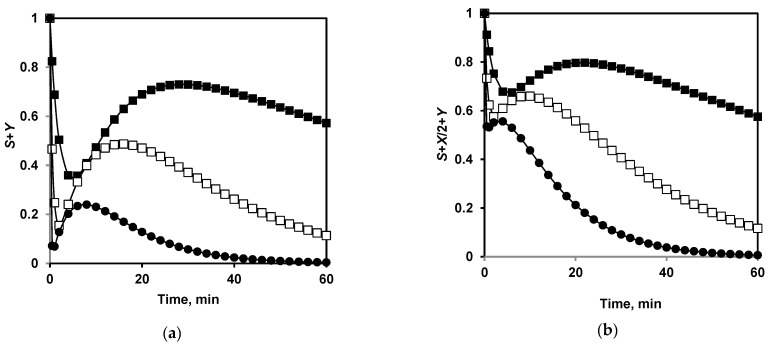
Prediction of the changes in secondary structures in course of proteolysis at different trypsin concentrations: *E*_0_ = 0.25 mg/L, *S*_0_/*E*_0_ = 1000 (▉); *E*_0_ = 1 mg/L, *S*_0_/*E*_0_ = 250 (□); and *E*_0_ = 4 mg/L, *S*_0_/*E*_0_ = 63 (●). (**a**) Secondary structures are assumed to be retained only in the original micelles *S* and nanoparticles *Y*. (**b**) It is assumed that secondary structures are half preserved in the hydrolyzed micelles *X.*

**Figure 11:**
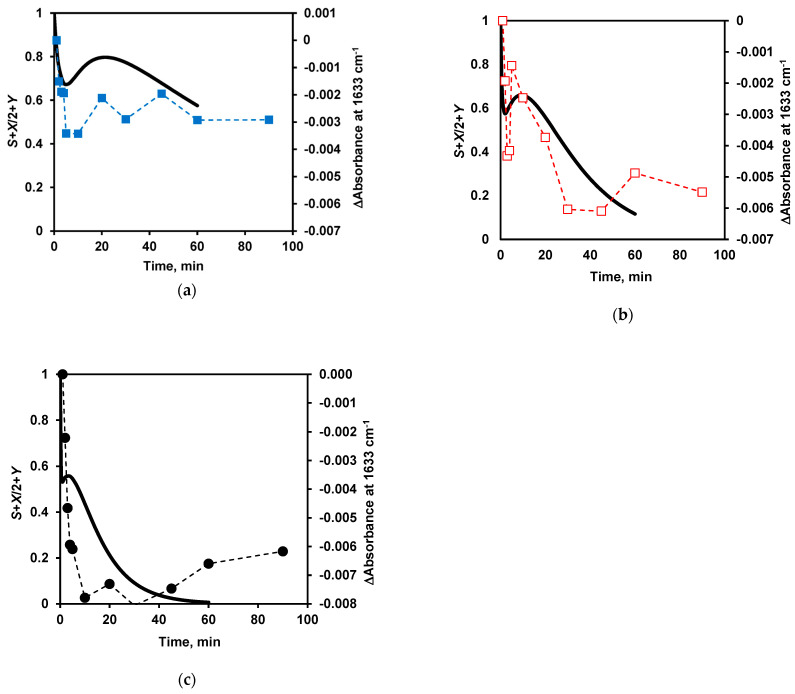
Comparison of the changes in secondary structures in the course of proteolysis according to the model (solid line) at *S*_0_/*E*_0_ ratios of 1000 (**a**), 250 (**b**), and 63 (**c**), and FTIR data (dotted line) at *S*_0_/*E*_0_ ratios of 10,000 (**a**, ■, blue), 4000 (**b**, □, red), and 500 (**c**, ●, black).

To evaluate the overall changes in the secondary structure during proteolysis, it is necessary to make assumptions about which compounds of the model (*S*, *X*, *Y*, or *N*) retain the secondary structure and which ones lose it as a result of the hydrolysis of peptide bonds. We considered two options: in the first, the secondary structures are only in the original micelles *S* and nanoparticles *Y*, so the content of secondary structures should be proportional to *S*+*Y* (Figure 10a) and in the second, it was assumed that in the addition to *S*+*Y* a half of secondary structures are preserved in the hydrolyzed micelles *X* and the overall content of secondary structures is *S*+*X*/2+*Y* (Figure 10b). The second case is more relevant to the experimentally observed curves for β-sheets, as shown in Figure 11. The same can be also demonstrated for α-helices.

The local maximum of the function *S*+*X*/2+*Y* (Figure 11) shifts towards shorter times as the *S*_0_/*E*_0_ ratio decreases, so that at the highest enzyme concentration (*S*_0_/*E*_0_ = 500) it becomes poorly distinguishable. This pattern is observed in the experimental dependences for both β-sheets and α-helices (Figure 5a,b).

## 3. Discussion

In the model proposed here, we did not focus on the sizes of particles, but rather on a small number of their fractions, which, we believe, have different properties. For these fractions, we imposed strict kinetic restrictions on the rate constants *k_I_*, *k_II_*, and *k_III_*, namely, their dependence or independence on *E*_0_, as well as the numerical values of these constants, which we obtained from the data of the light-scattering experiment. An assumption was also made about in which intermediate nanoparticles the secondary structure of the protein is retained, and in which it decreases. Then, we calculated what the concentration dependences for these fractions could be with a change in the concentration of the enzyme. To verify the model, we used FTIR data on the changes in the protein secondary structure, which provide important information for understanding proteolysis at the molecular level [29,30]. Despite its simplicity, the model correctly described the change in the content of β-sheets and α-helices during proteolysis with varying enzyme concentrations. As far as we know, this result was obtained for the first time.

The spectral region of the amide I bands that provide rapid knowledge on the protein secondary structures can be used to predict the hydrolysis of proteins and to follow the proteolysis process [29,30,40]. When constructing the model, it was assumed that *X* particles have a smaller proportion of secondary structures as a result of the hydrolysis of β-CN polypeptide chains, similar, for example, to thermal denaturation, which leads to a decrease in β-structures of self-assembling amphiphilic peptides [41]. We also used the assumption that the new particles *Y* have not a reduced, but have the same fraction of β-sheets as the original micelles *S*. Our previous work [30] revealed a temporary increase in β-sheets at the beginning of proteolysis at *S*_0_/*E*_0_ = 5000 compared to the non-hydrolyzed β-CN. The assumption that both fractions of the nanoparticles *X* and *Y* have a reduced number of β-sheets contradicts this experimental fact and does not give agreement between simulation results and FTIR data. A retention or even increase in β-sheets in the particles *Y* can be associated with the assembly of the amphiphylic β-CN polypeptides according to AFM data [22].

Aggregation of the unfolded proteins or the partially folded intermediates of proteins that undergo aggregation was found to give rise to the increased β-sheet signals and/or new β-sheet bands in the FTIR spectrum [42]. A similar effect was observed in the current study, with the difference that we did not consider the intact protein, but rather, a fraction of the intermediate component *Y* that was formed during proteolysis. In contrast to the generally accepted ideas about proteolysis, the proteolysis of β-CN by trypsin does not seem to be just a monotonous degradation of the secondary structure. It is important to note that this occurs at the low rates of peptide bond hydrolysis at *S*_0_/*E*_0_ = 10,000 or 4000.

To control the formation of peptide nanoparticles, it is convenient to use the method of static light scattering, in particular, the Debye method [43]. Since the nanoparticles are first formed during proteolysis and then destroyed by the same enzyme, there is a time interval when the maximum number of nanoparticles is formed. To determine this interval, the intensity of the light scattering at 45° was continuously measured directly in the reaction mixture with a time resolution of 1–2 min [24,25]. Thus, at different concentrations of the enzyme, we determined the times of particle formation (*t*_max_) at which the scattering intensities were maximum. This parameter is also important for the technology since it determines the time of adding the inhibitor in order to preserve the obtained nanoparticles.

The use of FTIR spectroscopy made it possible to determine the changes in the secondary structure during the rearrangement of particles. However, this method requires significantly higher sample concentrations (25 g/L) than those suitable for the light-scattering method (0.25 g/L), from which the kinetics was modeled. Therefore, for a correct comparison of the patterns of particle rearrangement in the proteolysis processes at concentrations of 25 g/L and 0.25 g/L, the different ranges of the *S*_0_/*E*_0_ ratio were used. For FTIR measurements, the *S*_0_/*E*_0_ ratios were 500, 4000, and 10,000, while for the proteolysis experiments in which *t*_max_ were measured, the corresponding ratios were 63, 250, and 1000. In our previous study [25], this problem was partially solved by comparing FTIR and light-scattering data obtained at different substrate concentrations, not at the same hydrolysis times, but at the same degrees of hydrolysis of peptide bonds.

In the model calculations for proteolysis of micellar β-CN at *E*_0_ = 0.25 mg/L, the rate of the hydrolysis of particles *Y* (0.0002 s^−1^) was significantly less than the rate of the hydrolysis of initial substrate *S* (0.0066 s^−1^). The rate of the third step (0.0002 s^−1^) was also less than the rate of the second one (0.0015 s^−1^) [24]. This can be explained by a decrease in specific peptide bonds in *Y* nanoparticles, since they are mainly hydrolyzed at the initial stage, as well as by an increased density of these nanoparticles [23], which can prevent the enzyme from penetrating into them.

It has been established that various peptide bonds in β-CN are hydrolyzed by trypsin with different rates, and the quantitative methods for the measurement of the corresponding kinetic parameters have been proposed [19,44]. However, using various hydrolysis rate constants would lead to overly complex equations containing these parameters. Therefore, in this work, only two hydrolysis rate constants, *k*_1_ and *k*_3_, were used. During hydrolysis with trypsin, the hydrophobic regions of the polypeptide chain of β-CN are not intensively hydrolyzed [19,24]. Our simple model takes into account the possibility of the formation of new nanoparticles based on the preserved hydrophobic centers. In this way, the model takes into account the specificity of the action of trypsin. In a more complex model, it is necessary to describe the kinetics of cleavage of the polypeptide chain regions capable of providing self-assembly processes and to use many more hydrolysis rate constants.

The formation of the particles during partial enzymatic hydrolysis of the proteins is not such a rare case [45,46]. However, in almost all cases, these processes are considered qualitatively and there is no quantitative model. We strongly believe that our model will also be useful for describing the formation of particles from the proteins in other cases.

## 4. Materials and Methods

### 4.1. Materials

β-CN (C6905) from bovine milk and trypsin from bovine pancreas were purchased from Sigma-Aldrich (St Louis, MO, USA). Trypsin (T1426) was treated with N-tosyl-L-phenylalanine chloromethyl ketone (TPCK) to inhibit chymotrypsin activity. Phosphate buffer solution was prepared with Milli-Q water and stored at 4 °C prior to use. Fresh trypsin solutions were prepared by diluting trypsin in phosphate buffer and used within one hour. All other reagents were of analytical grade obtained from commercial sources.

### 4.2. Proteolysis Reaction and Preparation of the Samples for AFM

The β-casein substrate was prepared by dissolving β-CN in 50 mM phosphate buffer (pH 7.9) at 37 °C with gentle stirring for at least 3 h. The reaction time for proteolysis in a volume of 10 mL at a concentration of *β*-CN S_0_ = 3.0 g/L was counted after adding and rapidly mixing 10 μL of trypsin stock solution (1 g/L) to provide trypsin concentration in the reaction mixture E_0_ = 1 mg/L. The proteolysis reaction was conducted at 37 °C with slight stirring and stopped either by heat treatment at 95 °C for 10 min (sample 1) or by adding a soybean trypsin inhibitor with trypsin to inhibitor ratio of 1:3 by weight (sample 2). The course of proteolysis was controlled by determining the degree of hydrolysis of peptide bonds (DH) by the OPA method [47].

### 4.3. Atomic Force Microscopy

The obtained nanoparticles were analyzed by atomic force microscopy using a FemtoScan microscope (Moscow, Russia) [23,27,48]. Samples 1 and 2 were diluted 100 times with water (Milli-Q), then aliquots of each diluted sample (2.5 μL) were dropped onto the freshly cleaved surface of mica and dried in air. The samples were analyzed at ambient conditions in the tapping mode of AFM. The Mikromasch cantilevers (resonant frequency of about 325 kHz, tip curvature radius of about 10 nm) were used. The 4 × 4 µm^2^ images were scanned to measure particle sizes. FemtoScan Online software [48] was used for AFM data processing and quantitative analysis. The surface sections were performed along the fast scanning axis in order to measure the diameters and heights of the nanoparticles.

### 4.4. FTIR Spectposcopy

β-CN and trypsin were prepared in 20 mM potassium phosphate buffer at pD 7.9 (in deuterated buffer) and were equilibrated overnight at +4 °C. Afterwards, equal volumes of substrate (β-CN) and enzyme (trypsin) stock solutions were mixed in a vial to initiate the enzymatic reaction. The β-CN concentration in the proteolysis reaction was 25 g/mL and trypsin concentrations were 0.05, 0.00625, and 0.0025 g/L. Thus, the β-CN to trypsin ratios (*S*_0_:*E*_0_ ratios) were 500:1, 4000:1, and 10,000:1 (*w*/*w*).

Measurements were performed with the FTIR Spectrometer (Perkin Elmer, UATR Two) having a DTGS detector in the transmission mode. A 2 μL sample from the proteolysis reaction was placed between demountable thin layer calcium fluoride windows, as described in our previous works [29,30]. The path length of the IR-cuvette was 14 μm. The spectra were recorded in the range of 4000–1000 cm^−1^ with a resolution of 4 cm^−1^ and 8 scans for 90 min at various time intervals. A circulating water system was used to keep the sample temperature constant at 37 °C during measurements. The air spectrum was recorded as background.

The FTIR spectral processing and visualization, and FTIR-difference spectra as well as quantitative data for intensity differences were carried out with ‘OPUS 7.0’ software (Bruker, Germany). As also described in our former works [29,30], firstly, the corresponding enzyme solution was subtracted from the β-CN-trypsin reaction mixture. For baseline-correction, the straight lines were interpolated between the points of the spectrum at 1725 cm^−1^ and 1375 cm^−1^ and they were subtracted from the spectrum. Afterwards, the spectra were normalized for equal area between 1725 cm^−1^ and 1375 cm^−1^. Finally, the FTIR-difference spectra were calculated by the subtraction of the first spectrum recorded at t = 1 min from each of the absorbance spectra recorded during enzymatic reaction. The intensity changes detected at 1593 cm^−1^ (antisymmetric stretching of free carboxylates as proteolysis products), 1650 cm^−1^ (α-helix), and 1633 cm^−1^ (β-sheet) obtained from the FTIR-difference spectra were plotted as a function of time in the course of enzymatic reaction for the *S*_0_/*E*_0_ ratios of 500, 4000, and 10,000.

## Data Availability

The data presented in this study are available on request from the corresponding author.
